# Supplementary material for: From cis-Lobeline to trans-Lobeline: Study on the Pharmacodynamics and Isomerization Factors
Source: Molecules. 2022 Sep 22;27(19):6253. doi: 10.3390/molecules27196253 (PMC9573392; doi:10.3390/molecules27196253)
Supplement: Supplementary file 1 [file molecules-27-06253-s001.zip › molecules-1889787-supplementary.pdf]

Supplementary Materials File S1.

From *cis*-Lobeline to *trans*-Lobeline: Study on the Pharmacodynamics and Isomerization Factors

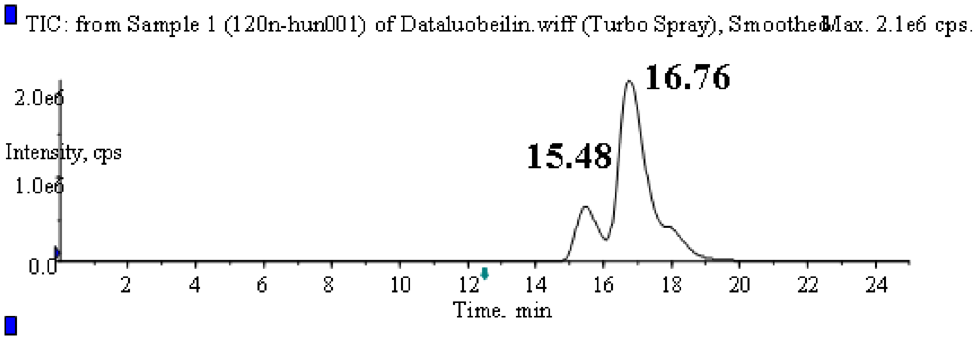

Figure S1. LC-MS/MS chromatogram of lobeline

Retention Time: *cis*-lobeline 16.76 min, *trans*-lobeline 15.48 min.

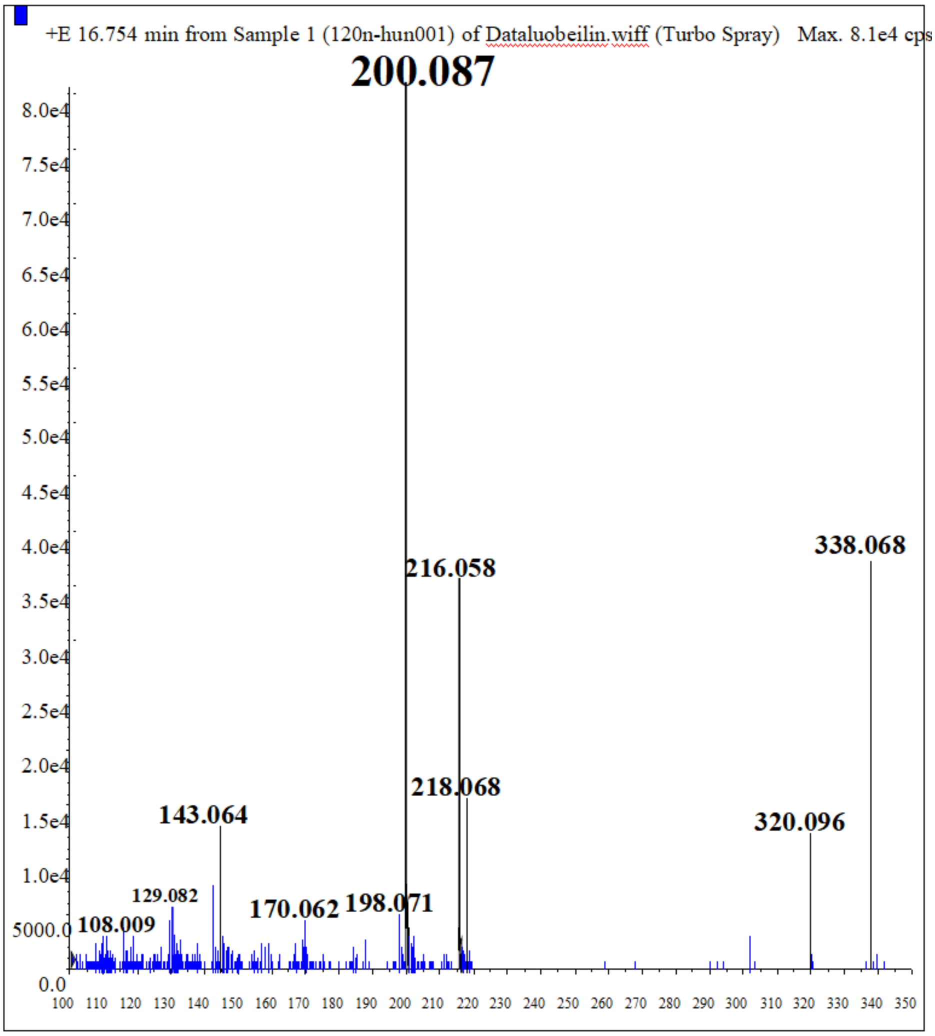

Figure S2. Secondary mass spectrum of *trans*-lobeline

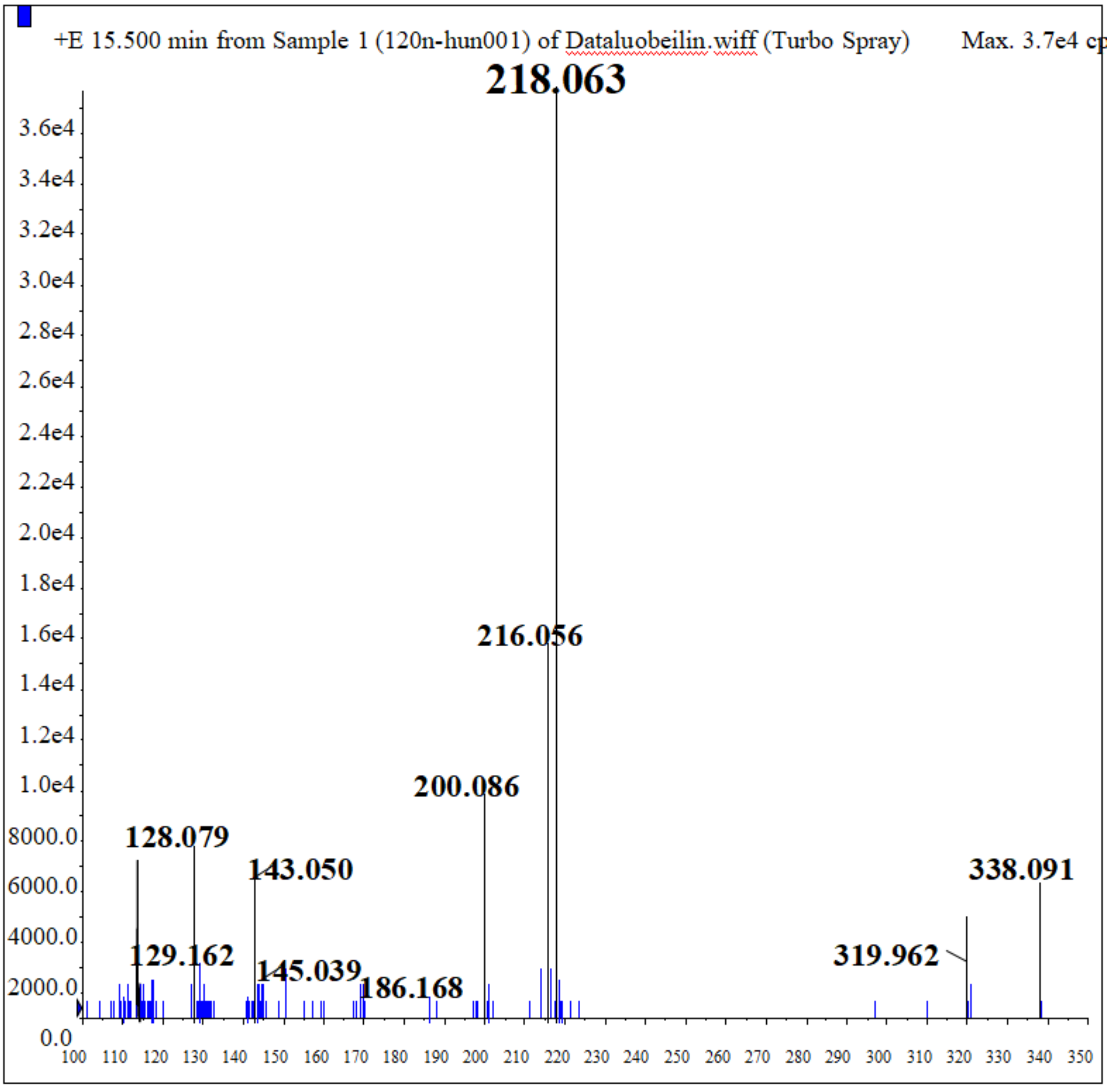

Figure S3. Secondary mass spectrum of *cis*-lobeline

Table S1. Fragment of *cis*-lobeline

Sample	m/z	Source	Fragment	Relative Intensity
cis-lobeline	338.06	$M+1^+$	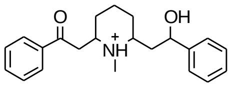	47%
	320.09	$M+1^+$-18	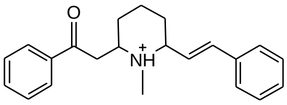	15%
	218.06	$M+1^+$-120	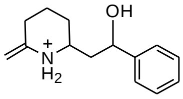	19%
	216.05	$M+1^+$-122	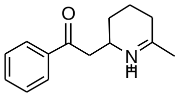	43%

	200.08	M+1$^+$-138	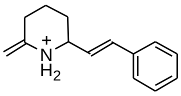	100%
	143.06	M+1$^+$-195	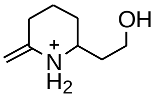	15%

Table S2. Fragment of *trans*-lobeline

Sample	m/z	Source	Fragment	Relative Intensity
trans-lobeline	338.09	M+1+	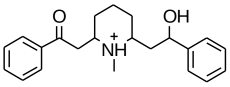	14%
	319.9	M+1+-18	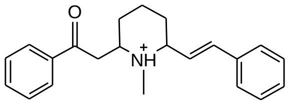	11%
	218.06	M+1+-120	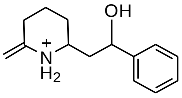	100%
	216.05	M+1+-122	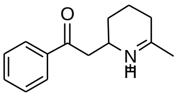	42%
	200.08	M+1+-138	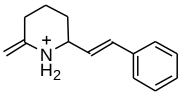	25%
	143.05	M+1+-195	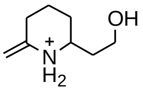	14%
	128.07	M+1+-210	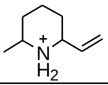	19%

According to the different retention time, the parent ion part has the same (or similar) structure, the base peak of the secondary mass spectrum is different, and the abundance ratio of each product ion confirms that in addition to *cis*-lobeline, there is also *trans*-lobeline in the lobeline solution.
